# The science of consciousness does not need another theory, it needs a minimal unifying model

**DOI:** 10.1093/nc/niaa013

**Published:** 2020-07-11

**Authors:** Wanja Wiese

**Affiliations:** Department of Philosophy, Johannes Gutenberg University, Jakob-Welder-Weg 18, 55128 Mainz, Germany

**Keywords:** theories and models, consciousness, minimal unifying model, free-energy principle, predictive processing, information generation

## Abstract

This article discusses a hypothesis recently put forward by Kanai *et al.*, according to which information generation constitutes a functional basis of, and a sufficient condition for, consciousness. Information generation involves the ability to compress and subsequently decompress information, potentially after a temporal delay and adapted to current purposes. I will argue that information generation should not be regarded as a sufficient condition for consciousness, but could serve as what I will call a “minimal unifying model of consciousness.” A minimal unifying model (MUM) specifies at least one necessary feature of consciousness, characterizes it in a determinable way, and shows that it is entailed by (many) existing theories of consciousness. Information generation fulfills these requirements. A MUM of consciousness is useful, because it unifies existing theories of consciousness by highlighting their common assumptions, while enabling further developments from which empirical predictions can be derived. Unlike existing theories (which probably contain at least some false assumptions), a MUM is thus likely to be an adequate model of consciousness, albeit at a relatively general level. Assumptions embodied in such a model are less informative than assumptions made by more specific theories and hence function more in the way of guiding principles. Still, they enable further refinements, in line with new empirical results and broader theoretical and evolutionary considerations. This also allows developing the model in ways that facilitate more specific claims and predictions.

**Highlights**

- The notion of a “minimal unifying model of consciousness” is introduced.
- Minimal unifying models characterize widely accepted, necessary properties of most conscious experiences.
- Minimal unifying models characterize these properties in a determinable way.
- It is argued that a model of information generation should be regarded as a minimal unifying model, not as a theory of consciousness providing sufficient conditions for consciousness.

## Introduction

In their article “Information generation as a functional basis of consciousness,” Kanai *et al.* (2019) suggest that information generation might constitute the functional basis of consciousness. Information generation, as defined in the article, roughly corresponds to the ability to (i) encode information in a format that allows the system to transiently keep the information in memory, in compressed form, and (ii) decompress that information to construct detailed representations (for online or offline processing). Empirically, this claim is supported by studies showing that consciousness requires the ability to keep information in (working) memory. Theoretically, it dovetails nicely with existing theories of consciousness. For instance, it is reminiscent of Gerald Edelman’s notion of a “remembered present” (Edelman 1989). Although Kanai *et al.* (2019) do not provide a formal definition of information generation, it also resonates with formal approaches to consciousness, such as Tononi *et al.*’s (2016) integrated information theory (Tononi), Thagard’s and Stewart’s (2014) semantic pointer competition theory, Ruffini’s (2017) Kolmogorov theory (KT), or van Hateren’s (2019) inversed-fitness-estimate theory.

Here, I will discuss the way Kanai *et al.* (2019) describe the status of their own hypothesis. While their main claim is that information generation is *necessary* for the functional roles associated with consciousness, they also propose that a stronger hypothesis should be taken into consideration, according to which information generation is *sufficient* for consciousness. If this is correct, then information generation, conceived as a teleological function, is entailed by consciousness; conceived as a functional mechanism, information generation is even sufficient for consciousness (Kanai *et al.* 2019, 8). [A mechanism for information generation would then be a mechanism that could explain why different types of evidence for phenomenal consciousness, such as verbal and non-verbal reports, the ability for planning and goal-directed action, etc., typically converge. The reason would be that there is a single mechanism underlying the different abilities that yield evidence for the presence of phenomenal consciousness (see Shea 2012). Of course, this presupposes that the different lines of evidence in fact do converge (*contra*Irvine, 2017).] Although the authors note that this hypothesis may strike many as implausible, they are ready to embrace the possibility that any system engaged in information generation, e.g. a variational autoencoder, is a conscious system (Kanai *et al.* 2019, 5). I will argue that a more plausible, and more fruitful hypothesis is that information generation can serve as what I will call a “minimal unifying model of consciousness.” (Strictly speaking, I am not referring to a model, but to a model *description* (see Weisberg 2007). A model description can, for instance, be given by a set of mathematical equations, but it can also describe the relevant properties of a concrete object, such as a scale model.) By a minimal unifying model of consciousness, I mean a model that

1. specifies only necessary properties of consciousness (i.e. it does not entail a strong sufficiency claim),
2. has determinable descriptions that can be made more specific, and
3. integrates existing approaches to consciousness by highlighting common assumptions.

A minimal unifying model (MUM) is thus minimal in a 2-fold sense: empirically, it is minimal because it only provides necessary conditions for consciousness; conceptually, it is minimal, because it characterizes these conditions in a general way. Still, it can be fruitful, because the characterization it offers can be specified in different ways, for instance, by developing formal definitions of properties characterized by the model. A MUM is unificatory by pointing to the “least common denominator” of existing accounts. All existing theories of consciousness can be expected to contain at least some false assumptions. A MUM, by contrast, seeks to identify assumptions shared by most approaches, thus highlighting the grains of truth that they have in common. [Although I would interchangeably say that a MUM *unifies* or that it *integrates* existing accounts, one could draw a distinction between unification and integration. Miłkowski (2016), for instance, characterizes explanatory unification as the project of finding general and simple explanations, whereas explanatory integration combines existing explanations. The project of developing a MUM aims at explanatory unification, not integration. A related approach is advertised by Graziano *et al.* (2019, 15), who argue that “[w]e may now have a […] family of theories that cohere and provide a working, mechanistic, scientifically meaningful, and even artificially buildable understanding of consciousness.” According to the authors, their own theory (attention schema theory, AST) “can be understood as a specific unification of GW [global workspace theory] and HOT [higher-order thought theory]” (2019, 13). The posits of AST overlap with other theories, and hence may provide a means of combining existing theories—however, this would amount to explanatory integration, not explanatory unification (in the sense of Miłkowski 2016). By contrast, a MUM seeks to abstract away from the dispensable parts of existing accounts, thereby offering a way of replacing existing theories (at least ideally).]

In what follows, I will first draw on a distinction between two types of consciousness: *structured* consciousness vs. *minimal* phenomenal experience. This will help to delimit the scope of a MUM and, more specifically, of accounts that associate consciousness with information generation. I will argue that only structured consciousness requires information generation, and, similarly, that a MUM will be most useful for accounts of structured consciousness. After that, I will review the notion of information generation, as characterized by Kanai *et al.* (2019), and will then argue that a (formal) model of information generation can serve as a MUM. Furthermore, I will explain why such a model is desirable, instead of a theory claiming to provide necessary *and* sufficient conditions for consciousness. In particular, I will justify the three characteristic features of MUMs of consciousness given above.

## Structured Consciousness and Minimal Phenomenal Experience

Most conscious waking states have structured contents: we typically experience many different objects at the same time and relations between them. Perhaps the most general way of describing this structure is in terms of space and time. Temporally, your conscious experience is structured in the sense that you experience events as happening *now*, which you can distinguish from past events that you consciously remember and from future events that you consciously anticipate. Furthermore, you consciously experience change: visually, you may perceive a bird flying toward its nest; aurally, you may perceive a bird song in which one chirping flows into the next. Spatially, your conscious experience is structured in the sense that you consciously perceive a space (perhaps a room in a building), and items within it (chairs, tables) that are connected by spatial distance relations and part-whole relations [as I argue in Wiese (2017), there are also experienced part-whole relations between experienced events]. Apart from spatiotemporal relations between contents of consciousness, conscious experience also typically has a subject–object structure. I experience everything from a subjective point of view: there is not just something it is like to have my experience, there is something it is like *for me* to have it (see Nagel 1974; Zahavi and Kriegel 2016).

I will call conscious experience with structured contents “structured consciousness” (see Ruffini 2017). We can contrast structured consciousness with the notion of “minimal phenomenal experience” (MPE). The concept of MPE was introduced by Windt (2015) and refers to the simplest possible type of conscious experience, which is sometimes described as *consciousness as such* or as *pure awareness*. More specifically, MPE is “atemporal, selfless, and not tied to an individual first-person perspective” (Metzinger 2020, 36). In particular, MPE does not have structured contents, although it may have unstructured content (Metzinger 2020, 38). That is, MPE is characterized by an absence of experienced spatial, temporal, or subject–object relations. Instances of MPE may occur, for instance, during dreamless sleep episodes (Windt *et al.* 2016), but it is also possible that MPE in fact underlies all conscious experiences (so it could even be present in instances of structured consciousness).

There are some commonalities between what I call a “minimal unifying model of consciousness” and the notion of MPE. If MPE underlies all types of experience, then MPE may have a unifying phenomenal character, and a model of MPE may provide a crucial building block for a complete theory of consciousness. However, it would not be a unifying model in the sense specified here, because it would not point to assumptions that most existing theories of consciousness have in common (this is simply because most existing theories are theories of structured consciousness, not of minimal phenomenal experience)—which constitutes a key difference to features implied by a MUM. Still, developing a model of MPE and developing MUMs can be seen as complementary strategies.

In what follows, I will focus on structured consciousness. Conscious experiences with structured contents require information generation, and a MUM will be most useful for accounts of structured consciousness (because most existing approaches focus on structured consciousness).

## What Is Information Generation?

In the first sections of their article, Kanai *et al.* (2019) characterize information generation as a teleological function, i.e. in terms of the purpose served by it. The purpose is to enable “non-reflexive behavior such as responding after a delay, or executing an action based on internally generated plans” (Kanai *et al.* 2019, 3). According to the authors, this requires “the ability to internally generate sensory representations that are not direct reflections of the current sensory input.” (Kanai *et al*. 2019, 3). Furthermore, tasks that (apparently) require conscious processing have in common that they presuppose the ability to make information available for cognitive subsystems *after a short delay* (see Kanai *et al.* 2019, 2–3). In conscious perception, for instance, information about a perceptual object must be flexibly available even when the object itself is not present anymore. This provides evidence for the hypothesis that information generation is a function of consciousness. As a next step, the authors suggest that what achieves this function is the computational process of internally generating (possibly) counterfactual representations. In addition, they highlight connections to, among others, reinforcement learning, predictive processing, and active inference.

Is information generation just the act of representing actual or counterfactual states of affairs? That would strike many as too liberal. As it turns out, the authors make a slightly more specific claim, by linking information generation to the process of “producing representations” using generative models (Kanai *et al.* 2019, 4), in a way that involves a “mapping from an abstract low-dimensional representation to a high-dimensional representation in the data (i.e. sensory) space” (Kanai *et al.* 2019, 5). In the brain, this may be implemented by feedback predictions (as suggested by predictive processing; see Hohwy 2013; Clark 2016; Wiese and Metzinger 2017), but purely feedforward implementations are possible as well (as an example, the authors discuss variational autoencoders, see Kanai *et al.* 2019, 4–5).

If we consider the hypothesized function of consciousness again, it seems that the essential part of this is that information is not just stored and, after a delay, reactivated, but that the information is first compressed and then becomes decompressed. Generative models are statistical models of the relation between sensory signals and their hidden causes, i.e. a generative model is a model of how sensory signals are generated. This suggests that “decompression” should be interpreted as a probabilistic computation, in which the uncompressed representation constitutes a (or the most) likely hypothesis, given the compressed information. In other words, it seems that decompression involves a probabilistic filling in of information that is not present in the compressed representation. In line with this, information generation should not be conceived of as a passive process of using an internal “mirror of nature” (Rorty 1979), but as an active process of producing representations that are based on stored information and have been adapted to current purposes.

This constitutes a difference to a related suggestion by Cleeremans (2005, 90): “*Stability* in time refers to how long a representation can be maintained active during processing. […] Stability of representation is clearly related to availability to consciousness, to the extent that consciousness takes time.” *Stability*, as characterized in the quoted passage, only requires that information be stored in a way that makes it available after a delay. Information generation, by contrast, also seems to require that the information be stored in a way that enables using it for different purposes (e.g. by compressing it), which means that further processing is necessary before the stored information can be used.

## What Type of Information Is “Generated” in Information Generation?

One could suspect that information generation does not really involve the *generation* of information: a decompressed representation does not contain any more information than was contained in its compressed form; it is only more redundant. To clarify in what sense information is generated by decompressing, it will be helpful to consider links between Kanai *et al.*’s (2019) account and Ruffini’s (2017) Kolmogorov theory (KT) of consciousness.

Kanai *et al.* (2019, 4) suggest that information generation in the brain involves *generative models*. The brain does not produce uncompressed representations in the way a personal computer decompresses a zip folder. Rather, an uncompressed representation is produced on the basis of a generative model. This generative model can, for instance, contain information about how internally generated actions will change the incoming flow of sensory signals (Adams *et al.* 2013). This allows the brain to anticipate sensory signals during action. A high-level representation of a motor intent constitutes a compressed representation, and a prediction of sensory signals, derived in accordance with a generative model, constitutes a decompressed representation. Crucially, the way action affects sensory signals is context-sensitive, which already points to one sense in which information is *generated*: the generative model encodes context-sensitive information (Gandolla *et al.* 2014), ensuring that information about the context is reflected by the process of decompression and in this sense contained in the decompressed representation (whereas it was not contained in the compressed representation).

Ruffini’s (2017) KT can help to further clarify the importance of (generative) models. According to KT, signals produced by the brain (e.g. motor commands or signals measured using EEG or MEG) during episodes of consciousness appear to be complex, but are produced by simple models (Ruffini 2017, 5). As a consequence, data streams produced by the conscious brain can be compressed. Formally, this means the *algorithmic complexity* (also called “Kolmogorov complexity”) of data produced by the brain appears to be high, but is in fact low. The algorithmic complexity of a string is the length of the shortest program that can generate the string (see Cover and Thomas 2006). A program that produces the string in question embodies an algorithm for producing the string, hence the name *algorithmic* complexity.

Recall that a generative model can be regarded as a model of how sensory signals are generated, and thereby specifies how to compute sensory signals from assumptions about their hidden causes. In other words, a generative model entails an algorithm for generating (predictions of) sensory signals. KT emphasizes the assumption that even seemingly complex sensory signals can be predicted using simple models. A general strategy to reduce complexity is to use a hierarchical (deep) model (which is highlighted by predictive processing and active inference; see Friston *et al.* 2018).

KT further suggests that there is a form of correspondence between sensory signals and data streams produced by conscious brains: the sensory input will be compressible to some extent, but, given the data produced by a conscious brain in response to sensory signals, the sensory input will be even more compressible (formally, this means that the “mutual algorithmic information” between the input and the “response” of a conscious agent is high, see Ruffini 2017, 6).

These two aspects highlighted by KT, i.e. simple models and a correspondence between sensory signals and internally generated data streams, can be traced back to a more fundamental assumption, provided by Friston’s (2010) free-energy principle (FEP). According to FEP, the brain minimizes variational free energy, a quantity that can equivalently be expressed in different ways, one of which involves terms for *complexity* and *accuracy*. Consequently, by minimizing free energy, the brain maximizes accuracy and minimizes complexity. As Friston (2010) points out, minimizing complexity “ensures that no excessive parameters are applied in the generative model and leads to a parsimonious representation of sensory data” (Friston 2010, 131). In other words, a system that minimizes free energy uses simple models to generate seemingly complex data streams (e.g. predictions of sensory signals or adaptive action). Furthermore, by maximizing accuracy, a system maximizes the mutual information between sensory input and internal responses (ibid.).

Summing up, KT can help to further clarify the notion of information generation. FEP can, in addition to this, provide a fundamental framework within which more specific (formal) definitions of information generation could be developed.

## Is Information Generation Necessary or Sufficient for Consciousness?

**Figure 1.**
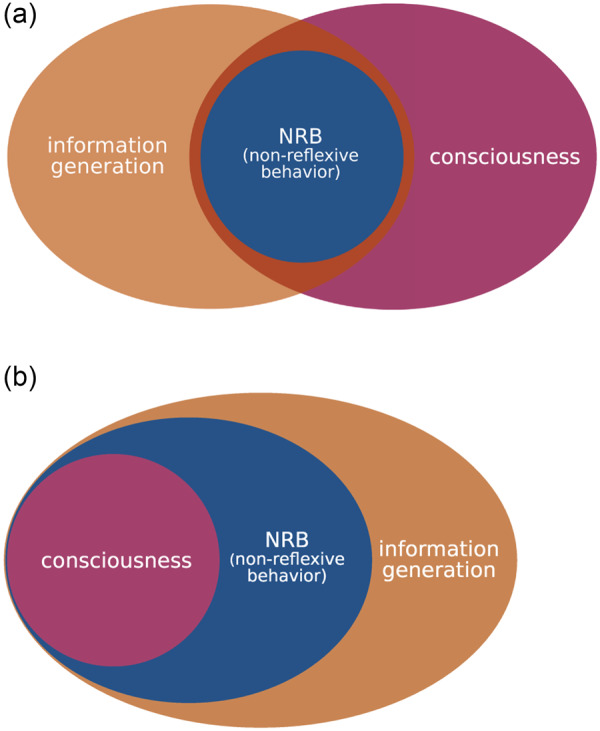
(a) Conceptually, NRB (such as “responding after a delay, or executing an action based on internally generated plans,” Kanai *et al.* 2019, 3) seems to require information generation. That is, if a system displays NRB, it is capable of information generation. Empirically, NRB seems to require consciousness (at least in human beings). That is, if a system displays NRB, it is conscious. These observations are compatible with the possibility of consciousness without information generation, and with the possibility of information generation without the capacity for NRB. However, as pointed out in the main text, there is reason to believe that information generation is necessary for consciousness. Furthermore, depending on how NRB is defined, it may not require consciousness. See (b) for an illustration. (b) An empirically informed, conservative view on the relationships between consciousness, NRB, and information generation: information generation is necessary for consciousness and NRB, but is not sufficient for either of them. That is, all conscious systems have the ability for information generation, and NRB without information generation is impossible. However, not all systems that generate information are conscious, and there may be forms of NRB that do not require consciousness. (Note that the main point of this figure is to illustrate the relationship between consciousness and information generation, as suggested by the treatment in the main text: information generation is necessary, but not sufficient for consciousness. The relationship between NRB and consciousness/information generation is only of peripheral importance. The main reason for this is that it largely is a terminological issue, i.e. it depends on how NRB is defined. Furthermore, it may be impossible to draw a sharp boundary between reflexive and non-reflexive behavior.)

What is the empirical evidence for the hypothesis that information generation is necessary for consciousness, and for the stronger hypothesis that information generation is sufficient for consciousness? Kanai *et al.* proceed by noting that there are cognitive capacities that seem to require consciousness: empirical results suggest that bridging a temporal gap in classical conditioning, a delayed response to perceptual stimuli, and planning are impossible without consciousness (or without conscious processing of the information in question). Hence, the hypothesis “that a key function of consciousness is to allow non-reflexive behavior such as responding after a delay, or executing an action based on internally generated plans” (Kanai *et al.* 2019, 3) is empirically plausible. This suggests that non-reflexive behavior (NRB) requires consciousness. Furthermore, it seems that diverse types of NRB all require information generation (in the sense discussed above). If consciousness is necessary for NRB, then NRB is sufficient for consciousness. Furthermore, if NRB requires information generation, then NRB is sufficient for information generation. But it does not follow that information generation is necessary for consciousness (let alone sufficient for consciousness). See Fig. 1a for an illustration. It is striking that many capacities that require consciousness also require information generation, but assuming an entailment relation between consciousness and information generation constitutes an additional step.

As intimated above, the idea that information generation is a central function of consciousness resonates with many existing theories of consciousness. By discussing relations to various existing approaches, Kanai *et al.* (2019, 6–7) highlight the importance of information generation for diverse phenomena such as regret, planning, the learning of causal relationships, perceptual presence, illusions, and dreaming. This provides evidence for the claim that information generation is necessary for consciousness (as noted in section “Structured consciousness and minimal phenomenal experience” above, this may not be true for minimal phenomenal experience). But it does not establish that information generation is sufficient for consciousness. As I will argue below, the hypothesis that information generation is necessary for consciousness should be regarded as the central contribution made by Kanai *et al.* (2019). The hypothesis that all forms of NRB entail consciousness is compatible with this hypothesis, but is not required (and may in fact be unnecessarily strong). The resulting view on the relationship between consciousness, NRB, and information generation is illustrated by Fig. 1b.

## What Is a MUM of Consciousness?

Here, I shall suggest that information generation can serve as a MUM of consciousness. A MUM specifies only necessary properties of consciousness, involves determinable descriptions that can be made more specific, and integrates existing approaches to consciousness. More specifically, a MUM is characterized by the following features:

1. Empirically, it is minimal by specifying properties that most states of consciousness have in common; i.e. it specifies only *necessary* features of (most) conscious experiences.
2. Conceptually, it is minimal by offering a *determinable* characterization of these properties that can be refined in various ways.
3. Furthermore, a MUM is *unifying* to the extent that it highlights assumptions that existing approaches have in common.

One could object that theoretical unification is completely unwarranted at this stage of the science of consciousness (but see Graziano *et al.* 2019). Rather than having a set of established and generally accepted theories, the science of consciousness is marked by competing theories that could, for all we know, turn out to be completely false. This is in stark contrast to the situation in, say, theoretical physics, in which quantum mechanics and general relativity are two generally accepted theories that do not compete, but account for different parts of reality (different forces). Hence, a unifying theory in theoretical physics (i.e. a theory of quantum gravity), is desirable. In contrast to this, the objection continues, the study of consciousness should not aim for unification, but should develop new, better theories, that make novel testable predictions and have advantages over existing theories. In particular, it would be desirable to have *more specific* theories, not a model that is *more general* than existing theories, and does not promote research by suggesting novel empirical predictions.

The objection ignores that theories of consciousness fulfill a dual role: first, they have to provide an empirically adequate (operationalized) definition of consciousness; secondly, they have to provide an explanation of consciousness, i.e. show how (and why) properties of physical systems give rise to conscious experience. The first role requires specifying the *explanandum* (i.e. what it is that a theory purports to explain); the second role requires specifying an *explanans* (i.e. a specification of the properties that account for the explanandum). The first role requires conceptual work (this is not to say that it *only* requires conceptual work, and not even that it requires *mainly* conceptual work; empirical results provide constraints on concepts of consciousness, and Kanai *et al.*’s article shows how empirical case studies can inform a conception of the functional basis of consciousness). The second role requires mainly empirical work. A MUM is primarily concerned with the first role. Furthermore, since a MUM is *determinable* (feature 2; see the following section for an explanation), it can be used to derive novel predictions, if further assumptions are added. As such, a MUM functions more like a framework than like a theory, because it allows more specific developments in different directions, that still conform to the same, overarching assumptions or guiding principles.

A potential additional virtue of this feature is that it may circumvent the problem that there is no general agreement on how to measure consciousness. Different criteria for the ascription of consciousness may come to diverging results in many cases (see Irvine 2017). Therefore, it can be useful to start with a determinable criterion that is necessary, but not sufficient for the ascription of consciousness (and hence less controversial). If indeed phenomenal consciousness is not a unique natural kind (*contra*Shea 2012), then a MUM could still be used to show what different kinds of phenomenal consciousness have in common.

In the next section, I will suggest that information generation satisfies the three requirements on MUMs, and will argue, against Kanai *et al.* (2019), that information generation should not be considered as being sufficient for consciousness.

## Information Generation as a MUM of Consciousness

In claiming that information generation is necessary for consciousness, Kanai *et al.* (2019) agree with the first two requirements on MUMs, but they suggest that information generation should also be taken into consideration when it comes to sufficient properties. I have three reasons for seeing this differently.

First, the empirical support for the sufficiency claim is too weak to justify it. In particular, the claim is challenged by possible cases of working memory without consciousness (Rosenthal *et al.* 2010; Soto *et al.* 2011; Trübutschek *et al.* 2017). While the debate about this is ongoing (Persuh *et al.* 2018; Nakano and Ishihara 2020), it nevertheless shows that the claim stands on empirically shaky feet.

Secondly, even if most states of consciousness can be described as a particular type of information generation, saying that consciousness is information generation is almost empty (just as the claim that consciousness can be described as inference, cf. Friston 2018). Arguably, a substantial version of the claim that consciousness is information generation must at least make a connection to (potential) action (see Seth 2009; Morsella *et al.* 2016; Pennartz 2017). For instance, a variational autoencoder is not capable of generating actions, and this alone may suffice to reject the claim that information generation implemented by such a system gives rise to consciousness. More generally, information generation should be regarded as a determinable property that itself is not sufficient for consciousness—although specific ways of generating information (i.e. some determinates of the determinable *information generation*) could be sufficient for consciousness. In general, a *determinable* is a property with respect to which more specific properties exist. These more specific properties are called *determinates* (Wilson 2017). For instance, *red* and *blue* are two different determinates of the same determinable *color*. Being blue is a particular way of being colored. Note that blue is a *determinate* with respect to *color*, but it is a *determinable* with respect to more specific properties, such as *azure* or *cyan*. In the context of a MUM, this means that a determinable such as *information generation* can not only be specified in different ways, but a given specification can, subsequently, be further specified. That is, the project of providing more specific models can be carried out step by step, yielding ever more determinate specifications of properties such as *information generation*.

Thirdly, focusing on the sufficiency claim distracts from the commonalities with existing theories of consciousness. Since all current theories are likely to contain at least some false assumptions, it will be more fruitful to consider to what extent information generation is *entailed* by existing theories. Identifying common assumptions is, I submit, more likely to track true assumptions than positing yet another theory of consciousness.

In line with Seth (2016), I have elsewhere (Wiese 2018a) argued that the *real problem* of consciousness consists in explaining all characteristic features of consciousness (such as global availability, information integration, temporal information). A challenge is created by the fact that a collection of features does not necessarily point to a unified concept of consciousness. Since measures of consciousness can come to different results in many cases, one could doubt that there is a single phenomenon, i.e. *consciousness*, that can be captured by a single scientific concept (see Irvine 2017). My proposal in Wiese (2018a) is to seek the help of a formal characterization of consciousness, based on as little assumptions as possible, which may then be shown to entail characteristic features of consciousness, given further, more specific assumptions (see also Wiese 2018b). For instance, minimizing (expected) free energy in deep models (which have temporal thickness) is a process that already entails many features associated with consciousness, although it is not sufficient for consciousness (*contra*Friston 2018). Similarly, a formal model of information generation could help to develop more specific formal characterizations that entail more features of consciousness (possibly even using the framework provided by Friston’s free-energy principle; cf. the remarks in the section “What type of information is ‘generated’ in information generation?” above). Even if it should turn out that phenomenal consciousness is not a unique natural kind, it could still be possible to regard different kinds of phenomenal consciousness as different types of information generation. Furthermore, information generation as a MUM of consciousness could help to discover the evolutionary basis of consciousness, e.g. by considering the relationship between information generation and what Ginsburg and Jablonka (2019) call the “evolutionary transition marker of consciousness” (which they propose to identify with unlimited associative learning).

## Conclusion: The Science of Consciousness Is in Its Infancy, It Needs a MUM

Kanai *et al.* (2019) make the compelling claim that information generation could serve as a functional basis for consciousness. In contrast to what the authors claim, I have argued that information generation should not be considered as a sufficient condition for consciousness (i.e. it is very likely that information generation is possible without consciousness). Instead, I suggested that information generation could serve as a MUM of consciousness. A MUM specifies at least one necessary feature of consciousness, characterizes it by making as little further assumptions as possible, and shows that it is entailed by (many) existing theories of consciousness. The claim that the feature is necessary must be justified by empirical studies, showing that (i) the absence of this feature goes along with unconscious processing, and (ii) functional roles associated with consciousness require this feature. Information generation, as illustrated using the variational auto-encoder, satisfies these requirements. It can therefore be regarded as a MUM. Having a MUM is useful, because it unifies existing theories of consciousness by highlighting their common assumptions, while enabling further developments from which empirical predictions can be derived. Unlike existing theories (which probably contain at least some false assumptions), a MUM is thus likely to make true assumptions about consciousness. These assumptions may be less informative than assumptions made by more specific theories, and hence function more in the way of guiding principles. Still, this enables further refinements, in line with new empirical results and broader theoretical and evolutionary considerations. Furthermore, this also allows developing the model in different ways that facilitate more specific claims and predictions. Hence, having a MUM is likely to constitute a key step in developing a mature science of consciousness, and, eventually, a complete and adequate theory of consciousness.
